# Identification of Neuroprotective Factors Associated with Successful Ageing and Risk of Cognitive Impairment among Malaysia Older Adults

**DOI:** 10.1155/2017/4218756

**Published:** 2017-10-03

**Authors:** Huijin Lau, Arimi Fitri Mat Ludin, Nor Fadilah Rajab, Suzana Shahar

**Affiliations:** ^1^Biomedical Science Programme, School of Diagnostic and Applied Health Sciences, Faculty of Health Sciences, Universiti Kebangsaan Malaysia, Kuala Lumpur, Malaysia; ^2^Dietetics Programme, School of Healthcare Sciences, Faculty of Health Sciences, Universiti Kebangsaan Malaysia, Kuala Lumpur, Malaysia

## Abstract

The increase of ageing population has raised public attention on the concept of successful ageing. Studies have shown that vitamin D, telomere length, and brain-derived neurotrophic factor (BDNF) have been associated with cognitive function. Therefore, this study aimed to identify neuroprotective factors for cognitive decline in different ageing groups. A total of 300 older adults aged 60 years and above were recruited in this population based cross-sectional study. Participants were categorized into three groups: mild cognitive impairment (MCI) (*n* = 100), usual ageing (UA) (*n* = 100), and successful ageing (SA) (*n* = 100). Dietary vitamin D intake was assessed through Diet History Questionnaire (DHQ). Out of the 300 participants, only 150 were subjected to fasting blood sample collection. These samples were used for serum vitamin D and plasma BDNF measurements. Whole blood telomere length was measured using RT-PCR method. The results show that the reduction of the risk of MCI was achieved by higher serum vitamin D level (OR: 0.96, 95% CI: 0.92–0.99, *p* < 0.05), higher plasma BDNF level (OR: 0.51, 95% CI: 0.30–0.88,  *p* < 0.05), and longer telomere (OR: 0.97, 95% CI: 0.95–0.99,  *p* < 0.001). In conclusion, participants with higher vitamin D level, higher BDNF level, and longer telomere length were more likely to age successfully.

## 1. Introduction

Ageing population in Malaysia is expected to reach 15% of the total population in year 2030 [1]. This can burden a nation especially that a significant percentage of healthcare budget is channelled to the elderly population. Recently, neurodegenerative diseases such as dementia and Alzheimer's diseases have become a major concern. The rapid growth of ageing population has raised much attention and awareness on the concept of successful ageing. The main goal of successful ageing is to maintain or improve emotional well-being and quality of life [2]. Therefore, discovery of potential noninvasive and easily accessible neuroprotective factors for cognitive impairment is needed to delay or prevent the onset of dementia and Alzheimer's disease. These would also promote successful ageing.

Balanced dietary intake is a well-established lifestyle factor in maintaining cognition during ageing. However, vitamin D deficiency is a very common problem in geriatric population. Previous animal study showed that vitamin D supplementation may reverse age-related cognitive decline in ageing rats [3]. In addition, cross-sectional study by Annweiler et al. [4] revealed that there is a significant association between vitamin D and cognitive function. Therefore, vitamin D has been suggested as a potential neuroprotective agent in ageing process.

Ageing is also often related to telomere shortening and decrease in BDNF level. Ageing increased oxidative stress, which leads to DNA damage characterized by telomere shortening [5]. Ma et al. [6] reported a significant correlation between telomere length and cognitive function and suggested that telomere length may serve as a biomarker for cognitive decline. BDNF level has been found to decrease with increasing age. High level of BDNF is believed to protect neurons from damage and maintain cognitive function in the elderly [7]. Therefore, reduction of BDNF could also be a risk factor for cognitive decline.

Although previous studies showed significant associations between the vitamin D level, telomere length, and BDNF level with cognitive function, their relationships with SA, UA, and MCI have not fully been explored. Therefore, this study aimed to identify the neuroprotective factors and their relationship with successful ageing and risk of MCI.

## 2. Method

This comparative cross-sectional study is a part of the large-scale community based longitudinal study which investigates neuroprotection model for the purpose of healthy longevity among Malaysian older adults (TUA) [8]. The study protocol was approved by Research and Medical Research Ethics Committee of Universiti Kebangsaan Malaysia (UKM) and informed consent was obtained from all the participants.

### 2.1. Participants and Cognitive Ageing Groups Classification

Participants in the previous large-scale study were divided into three different cognitive ageing groups, namely, mild cognitive impairment (MCI), usual ageing (UA), and successful ageing (SA). The classification of cognitive status was done based on multidimensional domains including physical function, subjective and objective memory impairments, psychocognitive functioning, major diseases, health status, and quality of life by using pretested questionnaires. The details of cognitive ageing groups classification protocol were described in Table 1 [8]. The present study involved a total of 300 community-dwelling older adults aged 60 years and above recruited from the previous study using multistage random sampling. A total of 100 participants with MCI were randomly drawn from the large population in the community based longitudinal study reported earlier [8]. Age- and gender-matched participants with UA (*n* = 100) and SA (*n* = 100) were randomly drawn from the same cohort. Sociodemographic information and dietary intake were obtained using questionnaire at this stage. Biological samples were randomly collected from 50 participants from each group.

#### 2.1.1. Sociodemographic Information and Dietary Intake of Vitamin D

Sociodemographic information including age, ethnicity, years of education, marriage, and living status, as well as alcohol intake and smoking habit, was recorded. Vitamin D intake was also recorded using Diet History Questionnaire (DHQ) among the total of 300 elderly. Standardised pictures were provided to guide the participants in filling the questionnaire. They were asked to recall all the foods and beverages consumed in the past 7 days from the day the questionnaire was administered. The obtained data were then analysed using Nutritionist ProTM diet analysis software (Axxya Systems, USA) to calculate the total vitamin D intake.

#### 2.1.2. Blood Samples Collection

A total of 150 age- and gender-matched subsamples were randomly chosen for laboratory analysis including serum vitamin D level, buccal micronucleus assay, absolute telomere length, and plasma BDNF level. A total of 5 ml of fasting blood sample was collected through venipuncture procedure by trained phlebotomist into three separate vacutainers. Total of 2 ml of blood samples was centrifuged for 10 minutes at 3500 r.p.m. to obtain plasma. Plasma and another 2 ml of whole blood were then stored at −80°C prior to analysis. The remaining 1 ml of whole blood was immediately sent to clinical lab for serum vitamin D level determination.

#### 2.1.3. Serum Vitamin D Level

The serum vitamin D level was measured using validated ARCHITECT 25-OH Vitamin D assay by BP clinical lab (BP Healthcare, Kuala Lumpur, Malaysia). It is a delayed one-step immunoassay including a sample pretreatment for the quantitative determination of vitamin D in human serum based on Chemiflex (CMIA technology with flexible) assay protocols. The resulting chemiluminescent reaction is measured as relative light units (RLUs). ARCHITECT i System optics (Abbott Laboratories, Wiesbaden, Germany) was used to detect an indirect relationship between the amount of vitamin D in the sample and the RLUs [9].

#### 2.1.4. Absolute Telomere Length

DNA extraction from whole blood was done using commercially available kit (QIAamp® Blood Mini Kit, QIAGEN, Netherlands). Absolute telomere length measurement was carried out based on method adopted from O'Callaghan and Fenech [10] using iQ5 Real-Time Polymerase Chain Reaction (RT-PCR) detection system (Bio-Rad, CA, USA). The standard oligomers used in this assay were as follows:

1. Telomere (TTAGGG)14
2. Single copy gene (SCG) 36B4 (CAGCAAGTGGGAAGGTGTAATCCGTCTCCACAGACAAGGCCAGGACTCGTTTGTACCCGTTGATGATAGAATGGG) (Bio Basics, Canada Inc.)

PCR primers included the following:

1. teloF (CGGTTTGTTTGGGTTTGGGTTTGGGTTTGGGTTTGGGTT),
2. teloR (GGCTTGCCTTACCCTTACCCTTACCCTTACCCTTACCCT),
3. 36B4F (CAGCAAGTGGGAAGGTGTAATCC)
4. 36B4R (CCCATTCTATCATCAACGGGTACAA) (Bio Basic, Canada Inc.)

Absolute telomere length was calculated by dividing kb/telomere reaction with copies/diploid genome of 36B4.

#### 2.1.5. Plasma Brain-Derived Neurotrophic Factor (BDNF)

Plasma concentration of BDNF was quantified using commercially available enzyme-linked immunoassay (ELISA) kit (Sigma Aldrich, USA) based on manufacturer's instructions. Duplicates of BDNF levels were determined at an absorbance of 450 nm. The coefficient of variation between the standards and duplicates was less than 5% [11].

## 3. Data Analysis

All data were analysed using Statistical Package for Social Science (SPSS) version 22. Significant value was set at *p* < 0.05. Comparison of sociodemographic factors and potential neuroprotective factors between SA, UA, and MCI groups were analysed using *χ*^2^ tests for categorical variables and one-way Analysis of Variance (ANOVA) test for continuous variables. Results are presented as mean ± standard deviation for normally distributed data and median (quartile range) for data that were not normally distributed. LSD post hoc test was used to compare the significant difference of continuous variables between groups. Multinomial Logistic Regression was performed to determine the significant neuroprotective factors related to successful ageing and cognitive impairment.

## 4. Results

Table 2 summarizes the sociodemographic characteristics of the 300 participants. The mean age of participants was 68.04 ± 5.56 years old, and there was no significant difference between ageing groups (*p* > 0.05). The majority of the participants in this study were Malays (61.3%), followed by Chinese (35.7%) and Indians (3.0%). A total of 67.7% participants received education less than 6 years and the rest received education more than 6 years. The difference between ageing groups is significant (*p* < 0.001). Cognitive performances, physical function, and depressive scale also showed significant difference between groups (*p* < 0.05). However, smoking habit and ethnicity among participants did not show significant difference in the three ageing groups.

**Figure 1:**
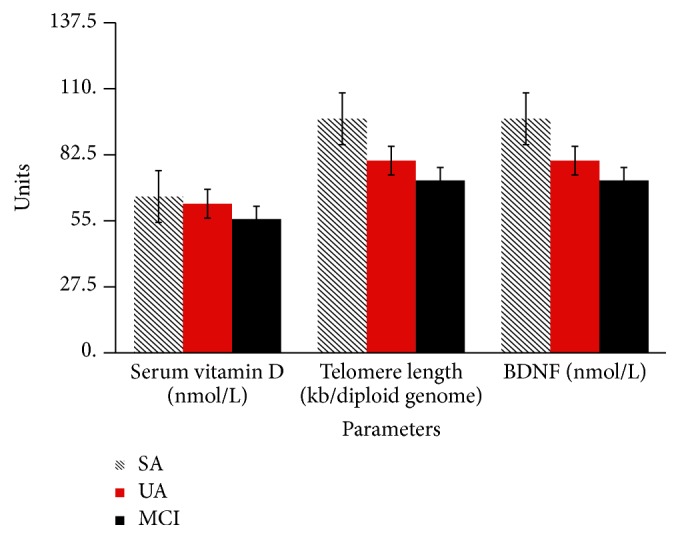
Comparison of selected neuroprotective factors between ageing groups. BDNF: brain-derived neurotrophic factor; SA: successful ageing; UA: usual ageing; MCI: mild cognitive impairment. One-way ANOVA and post hoc test LSD. Serum vitamin D and BDNF significant between MCI and SA; telomere length significant between UA and SA.

Figure 1 shows the comparison of selected neuroprotective factors between different ageing groups. Vitamin D intake was significantly higher in SA group (0.33 ± 0.77 *μ*g) compared to MCI group (0.13 ± 0.33 *μ*g) (*p* < 0.05). Participants in SA group also showed significantly higher serum vitamin D level (65.11 ± 17.08 nmol/L) than those in MCI group (55.71 ± 19.97 nmol/L) (*p* < 0.05). Telomere length was also reported to be significantly different between SA (97.52 ± 35.49 kb/genome diploid) with UA (80.07 ± 32.78 kb/genome diploid) and MCI (71.84 ± 30.97 kb/genome diploid) (*p* < 0.05). Plasma BDNF was reported to be higher in SA group (14.24 ± 1.26 nmol/L) than MCI group (13.38 ± 26.2 nmol/L) (*p* < 0.05).

The selected neuroprotective factors that were found to be significantly different (*p* < 0.05) were further analysed by Multinomial Logistic Regression model for the identification of significant neuroprotective factors associated with successful ageing and the risk of cognitive impairment.  Table 3 demonstrates the significant neuroprotective factors associated with the risk of cognitive impairment. After controlling the age, gender, educational years, and smoking status, it was found that every one  nmol/L increased in serum vitamin D level could reduce the risk of MCI by 4% (OR: 0.96, 95% CI: 0.92–0.99, *p* < 0.05). The risk of getting MCI and UA can also be lowered by 3% (OR: 0.97, 95% CI: 0.95–0.99, *p* < 0.001) and 2% (OR: 0.98, 95% CI: 0.96–0.99, *p* < 0.01), respectively, with an increase of one kb/genome diploid in telomere length when compared to SA group. In addition, increase in one  nmol/L in plasma BDNF level may also reduce the risk of MCI (OR: 0.51, 95% CI: 0.30–0.88,  *p* < 0.05) and UA (OR: 0.56, 95% CI: 0.33–0.93, *p* < 0.05) by 49% and 44%, respectively.

## 5. Discussion

Malnutrition such as vitamin D and protein deficiency accompanying the ageing process and living a sedentary lifestyle could accelerate muscle weakness. Increased prevalence of disability, falls, fractures, and even mortality among older adults is often related to frailty and sarcopenia. In a systematic review, most cross-sectional and longitudinal studies also demonstrate significant relationships between frailty and cognitive performance and impairment [12]. Therefore, a link between vitamin D deficiency and cognitive impairment could be hypothesized. Our results revealed that dietary intake and serum vitamin D levels were significantly higher in SA group than MCI group. In addition, the findings also showed that increased vitamin D level was associated with the risk of MCI. The wide distribution of vitamin D receptors (VDR) in central nervous system (CNS) has suggested that vitamin D plays a role in neurogenesis [13]. This finding is in line with previous study which reported that increased serum vitamin D could reduce the risk of MCI among the elderly [4]. Ahn and Kang [14] also stated in their study that serum vitamin D could be the predictor for cognitive decline based on MMSE score among the elderly. However, there was no significant association between serum vitamin D with MMSE score in the study conducted by Lapid et al. [15]. The optimum level of vitamin D to prevent bone fractures is between 50 and 80 nmol/L [16]. However, its optimum level to maintain cognitive function has not been identified.

In this study, the telomere length was found to be significantly longer in SA group as compared to MCI group. The significant association between longer telomere length and reduced risk of MCI among older adults is novel. Our finding corroborates Roberts et al. [17] which reported that shorter telomere length was significantly associated with MCI. In contrary, Hochstrasser et al. [18] did not observe any significant difference in telomere length between control and MCI group among older adults aged 70 years and above. Their finding was also confirmed by Arai et al. [19]. The inconsistency across studies might be attributed by several reasons. This includes narrow age range in the previous studies [5], wide inter- and intraindividual variability in telomere lengths, unmeasured confounding, and the use of different cognitive assessment tools (Mather et al. 2011). Nevertheless, the underlying mechanisms are still unclear even though the significant association was observed in this study. One possible explanation is that oxidative stress is a common cause of both telomere shortening and cognitive decline [20].

Serum contains large amount of platelets and has a longer lifespan of turnover, which is approximately 10 days. Plasma was chosen over serum in measuring BDNF in this study as it has a shorter lifespan of turnover as compared to serum due to minimal influence of the amount of platelets. In addition, vascular endothelial cells and the brain secrete the circulating BDNF in plasma [21]. Peripheral BDNF level could represent the BDNF brain level. These reasons suggested that plasma BDNF is more suitable to indicate the BDNF levels in the brain. Several animal studies demonstrated positive association between BDNF levels in frontal cortex and hippocampus with plasma BDNF levels. Positive correlation was also found between serum and cortical levels of BDNF [22]. It is thus suggested that BDNF in the central nervous system (CNS) may change along with the changes of in peripheral BDNF levels.

Higher plasma BDNF level was significantly associated with reduced risk of MCI in this study. Our finding is consistent with studies by Shimada et al. [23] and Turana et al. [24]. Generally, decreased BDNF levels in MCI may contribute to the development of neurodegenerative diseases such as dementia and Alzheimer's disease. These diseases may be due to lack of neurotrophic support. Although peripheral BDNF level was found to potentially act as protective factor for cognitive decline, the optimum level of peripheral BDNF is yet to be determined. Several studies have identified significant contrasting differences in BDNF levels between healthy and MCI/dementia/Alzheimer's disease (AD) participants. Lee et al. [25] observed an increased BDNF levels with MCI and Alzheimer's disease. However, Borba et al. [26] reported a decrease BDNF levels in MCI and dementia participants when compared to healthy controls. Decreased plasma BDNF levels were related to depression, bipolar disorder, and anxiety [27], whereas increased BDNF levels were found to be associated with smoking [28]. Therefore, factors such as depression, smoking, and alcohol intake should be taken into consideration as these could affect the BDNF levels and contribute to contrasting findings.

This study has several limitations. Firstly, the division of the ageing groups (SA, UA, and MCI) may raise conflict on the merging between social and clinical concepts of ageing. This is because, to date, there is no standard clinical diagnostic procedure to determine successful ageing or normal ageing. Achievement of successful ageing presented here may not be realistic for most people as stated by Bowling and Dieppe [29]. Some older people who do not have any diseases or impairments may not consider themselves as successful agers. However, there is also evidence that many older adults consider themselves to be happy and satisfied with life, despite the illness or physical function decline that they have. Secondly, it is possible that some of the potential confounders for vitamin D level determination were not measured in this study. These include parathyroid hormone, hours of sunlight exposure, the use of sunscreen products, and outdoor activities. Despite all the limitations, our findings provide substantial insights into the potential of vitamin D, telomere length, and BDNF as neuroprotective factors for MCI and their relationships with successful ageing.

## 6. Conclusion

In conclusion, vitamin D level, telomere length, and BDNF could act as neuroprotective factors for mild cognitive impairment. However, future prospective studies are warranted to determine the cut-off value of these factors in maintaining cognitive function among older adults. Besides, the relevant professionals should also establish a more balanced and interdisciplinary perspective on ageing due to the great heterogeneity that exists among the older adults.

## Figures and Tables

**Table 1:** Classification criteria for ageing groups.

Successful ageing	Usual ageing	Mild cognitive impairment
(1) Free from diabetes, hypertension, cancer, heart diseases, chronic lung diseases, and stroke	(1) Participants with average performance in most of the cognitive test administered (scores above MCI but below SA)	(1) Subjective memory complaint by participants or caregivers
(2) No functional limitations as indicated by full score in ADL	(2) No dementia	(2) Objective memory impairment [poor performance in one or more cognitive tests (Digit span and RAVLT) with score of at least 1.5 SD below the mean average]
(3) Normal global function as indicated by MMSE score ≥ 22	(3) No or very minimal functional limitations	(3) No dementia
(4) No depression by having a score of ≤4 in the GDS-15 item		(4) No limitations in basic activities of daily living (ADL)
(5) Good quality of life		(5) No or very minimal difficulties in instrumental activities of daily living by having a score of ≤1.5 SD from mean norm
(6) Good self-perceived health		(6) Preserved global function by having MMSE score of ≥19

ADL: activities of daily living; MMSE: Mini-Mental State Examination; GDS: Geriatric Depression Scale.

**Table 2:** Sociodemographic characteristics.

Parameter	SA (*n* = 100)	UA (*n* = 100)	MCI (*n* = 100)	Total (*N* = 300)	*p*
*Age* ^a^	67.99 ± 5.52	68.00 ± 5.57	68.14 ± 5.64	68.04 ± 5.56	>0.05
*Ethnic*					
Malay	61 (33.2)	61 (33.2)	62 (33.7)	184 (61.3)	>0.05
Chinese	36 (33.6)	36 (33.6)	35 (32.7)	107 (35.7)	
Indian	3 (33.3)	3 (33.3)	3 (33.3)	9 (3.0)	
*Marriage status*					
Single/widow/widower/divorced	17 (17.0)	19 (19.0)	25 (25.0)	61 (20.3)	>0.05
Married	83 (83.0)	81 (81.0)	75 (75.0)	239 (79.7)	
*Educational year*					
≤6 years	44 (44.0)	78 (78.0)	81 (81.0)	203 (67.7)	<0.001
>6 years	56 (56.0)	22 (22.0)	19 (19.0)	97 (32.3)	
*Smoking habit*					
Smoking	20 (20.0)	20 (20.0)	26 (26.0)	66 (22.0)	>0.05
Past or nonsmokers	80 (80.0)	80 (80.0)	74 (74.0)	234 (78.0)	
*Cognitive measures* ^a^					
MMSE	26.34 ± 0.22	23.24 ± 0.40	22.18 ± 0.48		<0.001^*∗*^
Digit Span	13.88 ± 0.34	11.96 ± 0.37	10.72 ± 0.39		<0.001^*∗∗*^
RAVLT	8.82 ± 0.29	7.28 ± 0.30	1.68 ± 0.13		<0.001^*∗∗*^
*Physical function* ^a^					
ADL	14.00 ± 0.00	12.42 ± 0.25	12.17 ± 0.17		<0.001^*∗∗∗*^
*Depressive symptoms* ^a^					
GDS	1.98 ± 0.14	2.07 ± 0.13	1.76 ± 0.13		>0.05
*Vitamin D intake *(*μg*)^a^	0.33 ± 0.77	0.25 ± 0.21	0.13 ± 0.33		<0.05

SA: successful ageing; UA: usual ageing; MCI: mild cognitive impairment; MMSE: Mini-Mental State Examination; ADL: activities of daily living; GDS: Geriatric Depression Scale. Chi-squared test, significant at *p* < 0.001. ^a^One-way ANOVA (mean ± standard deviation). ^*∗*^Significant at *p* < 0.05 between SA and MCI. ^*∗∗*^Significant at *p* < 0.001 between SA, UA, and MCI. ^*∗∗∗*^Significant at *p* < 0.001 between SA and UA, SA, and MCI.

**Table 3:** Determination of significant neuroprotective factors associated with risk of cognitive impairment.

	Selected neuroprotective factors	*B*	exp(*B*)	95% confidence interval		*p*
				Upper	Lower	
MCI	Serum Vitamin D (nmol/L)	−0.05	0.96	0.92	0.99	<0.05^a^
	Telomere Length (kb/diploid genome)	−0.03	0.97	0.95	0.99	<0.001^a^
	BDNF (nmol/L)	−0.67	0.51	0.30	0.88	<0.05^b^
UA	Serum Vitamin D (nmol/L)	−0.02	0.98	0.94	1.02	>0.05
	Telomere Length (kb/diploid genome)	−0.03	0.98	0.96	0.99	<0.01^a^
	BDNF (nmol/L)	−0.59	0.56	0.33	0.93	<0.05^b^

BDNF: brain-derived neurotrophic factor; SA: successful ageing; UA: usual ageing; MCI: mild cognitive impairment. Multinomial Logistic Regression with reference group is successful ageing. ^a^Significant at *p* < 0.05 after being controlled for age, gender, educational level, and smoking status. ^b^Significant at *p* < 0.05 after being controlled for confounding factors: age, gender, educational level, smoking, physical function, and depressive symptoms.
